# Supplementary material for: Learning to detect AI texts and learning the limits
Source: PLoS One. 2025 Oct 15;20(10):e0333007. doi: 10.1371/journal.pone.0333007 (PMC12527182; doi:10.1371/journal.pone.0333007)
Supplement: S2 File — All data and scripts used in the study, some additional descriptive statistics, scalable formate figures. (ZIP) [file pone.0333007.s002.tgz › data and scripts/OSF/scripts/analysis/Dependencies/GLS5_distance_vs_Confidence.pdf]

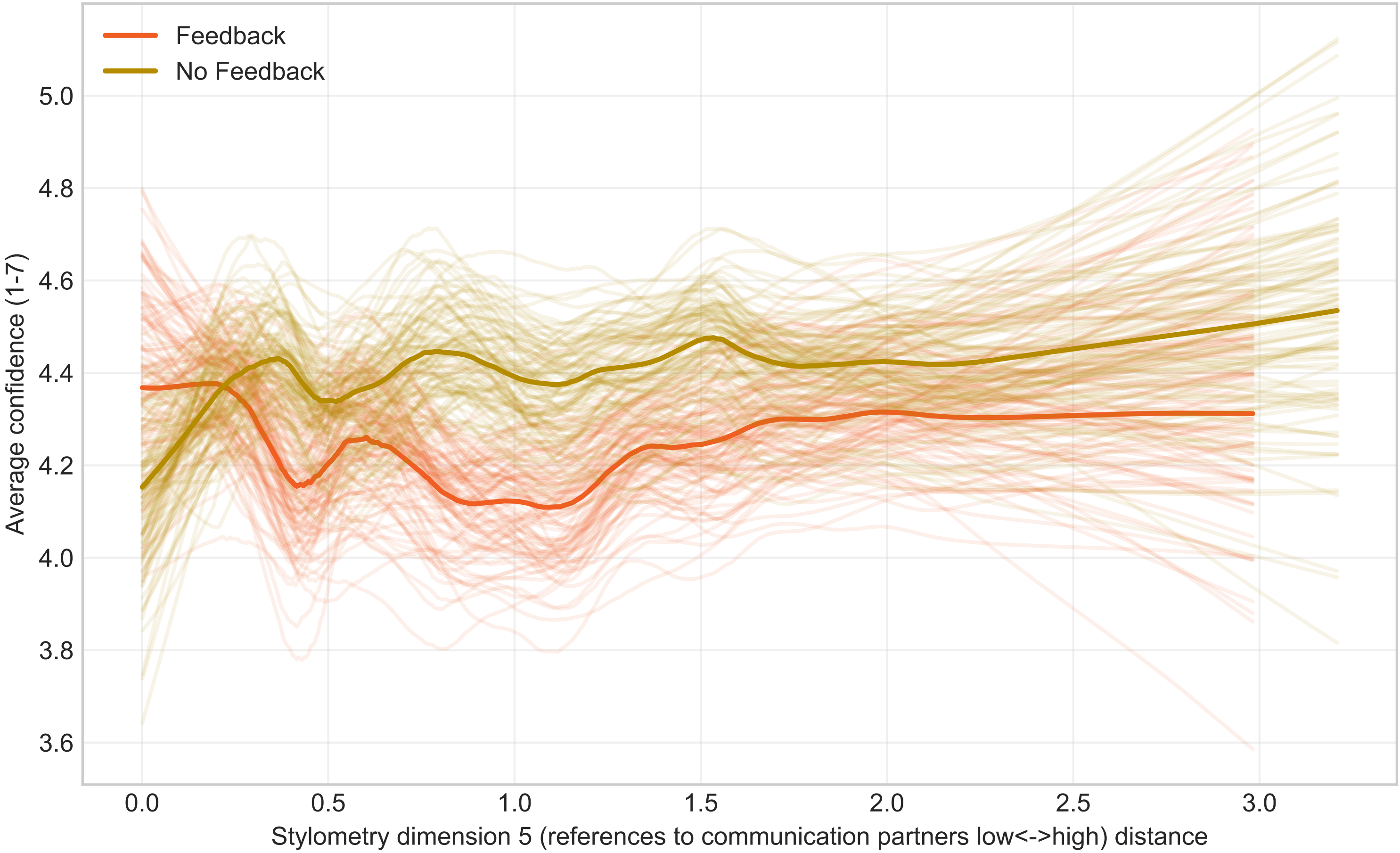
Feedback
No Feedback
Average confidence (1-7)
5.0
4.8
4.6
4.4
4.2
4.0
3.8
3.6
0.0
0.5
1.0
1.5
2.0
2.5
3.0
Stylometry dimension 5 (references to communication partners low<->high) distance
